# Predicting the duration of sickness absence due to knee osteoarthritis: a prognostic model developed in a population-based cohort in Sweden

**DOI:** 10.1186/s12891-021-04400-8

**Published:** 2021-07-02

**Authors:** Johanna Holm, Paolo Frumento, Gino Almondo, Katalin Gémes, Matteo Bottai, Kristina Alexanderson, Emilie Friberg, Kristin Farrants

**Affiliations:** 1 Division of Insurance Medicine, Department of Clinical Neuroscience, Karolinska Institutet, SE-171 77 Stockholm, Sweden; 2 Department of Political Sciences, University of Pisa, Via F. Serafini 3, 56126 Pisa, Italy; 3 Division of Biostatistics, Institute of Environmental Medicine, Karolinska Institutet, SE-171 77 Stockholm, Sweden

**Keywords:** Knee osteoarthritis, Sick-leave, Prediction, Sickness absence, Duration

## Abstract

**Background:**

Predicting the duration of sickness absence (SA) among sickness absent patients is a task many sickness certifying physicians as well as social insurance officers struggle with. Our aim was to develop a prediction model for prognosticating the duration of SA due to knee osteoarthritis.

**Methods:**

A population-based prospective study of SA spells was conducted using comprehensive microdata linked from five Swedish nationwide registers. All 12,098 new SA spells > 14 days due to knee osteoarthritis in 1/1 2010 through 30/6 2012 were included for individuals 18–64 years. The data was split into a development dataset (70 %, n_spells_ =8468) and a validation data set (n_spells_ =3690) for internal validation. Piecewise-constant hazards regression was performed to prognosticate the duration of SA (overall duration and duration > 90, >180, or > 365 days). Possible predictors were selected based on the log-likelihood loss when excluding them from the model.

**Results:**

Of all SA spells, 53 % were > 90 days and 3 % >365 days. Factors included in the final model were age, sex, geographical region, extent of sickness absence, previous sickness absence, history of specialized outpatient healthcare and/or inpatient healthcare, employment status, and educational level. The model was well calibrated. Overall, discrimination was poor (c = 0.53, 95 % confidence interval (CI) 0.52–0.54). For predicting SA > 90 days, discrimination as measured by AUC was 0.63 (95 % CI 0.61–0.65), for > 180 days, 0.69 (95 % CI 0.65–0.71), and for SA > 365 days, AUC was 0.75 (95 % CI 0.72–0.78).

**Conclusion:**

It was possible to predict patients at risk of long-term SA (> 180 days) with acceptable precision. However, the prediction of duration of SA spells due to knee osteoarthritis has room for improvement.

**Supplementary Information:**

The online version contains supplementary material available at 10.1186/s12891-021-04400-8.

## Significance and  innovation

- Judging the appropriate or likely duration of SA has been reported as one of the tasks clinicians find most problematic.
- We used comprehensive real-world data regarding sociodemographic and clinical factors for > 12,000 SA spells due to knee osteoarthritis to develop a prediction model for duration of SA.
- The developed prediction model included nine readily available factors.
- It was possible to discriminate long-term SA spells (> 365 days) with moderate precision, while shorter-term individual-level discrimination was lower.

## Introduction

Osteoarthritis of the knee is a common musculoskeletal diagnosis, with an age-standardized prevalence estimated at 3–4 % in the Nordic region, with an incidence heavily increasing with age [1]. Risk factors are largely genetic, but older age and overweight, as well as occupational exposures like kneeling are also important predisposing contributors [2–5]. With changing population demographics and the obesity epidemic, the prevalence of knee osteoarthritis is expected to continue to increase [6]. Between 1998 and 2015, the annual increase in the Swedish hospitalization rate of knee osteoarthritis exceeded 2 %. Only about a third of this increase was found to be explained by an increasingly aging population, thus hospitalizations due to knee osteoarthritis in the working ages are also rising [7]. It could also be expected that sickness absence (SA) certification due to knee osteoarthritis will remain a common task for treating physicians, as individuals with osteoarthritis are at almost twice the risk of SA as compared to the general population [8]. However, the task of prognosticating a SA spell is not a trivial one [9–13]. There is hardly any scientific knowledge on this; the few studies conducted show that neither physicians nor insurance agency professionals can accurately predict what patients are at risk of long-term SA, and physicians are mainly able to predict shorter-term SA with some accuracy [14–16]. Therefore, a clinical model for prognosticating the duration of a SA spell could potentially be useful as a decision-support tool, to aid identification of patients who are at risk of long-term SA and therefore are most likely to benefit from additional active rehabilitation efforts. Currently, there has not been any model developed to predict the duration of a SA spell due to knee osteoarthritis. In this study, we aimed to develop a parsimonious prediction model of SA spell duration due to knee osteoarthritis, to be used in the Swedish healthcare system.

## Materials and methods

Included were all 12,098 new SA spells lasting > 14 days with ICD-10 diagnosis code ’M17’ as primary diagnosis in Sweden during the 2.5-year period 2010.01.01 through 2012.06.30, among people aged 18–64 years when the SA spell started. If an individual had two or more SA spells, they were treated as independent events. SA spells were identified through the MiDAS database kept by the Swedish Social Insurance Agency.

### Framework: Sickness absence and disability pension benefits in Sweden

In Sweden, all residents above the age of 15 years with an income from work or unemployment benefits are covered by the public SA insurance. Individuals are eligible for SA benefits if their work capacity is reduced due to disease or injury [17]. A medical certificate is required from day eight. Day 1 is a waiting day, with no reimbursement. The employer reimburses income loss during days 2–14, after which SA benefits are administered by the Social Insurance Agency. For unemployed, the Social Insurance Agency issues reimbursements from day 2. The Social Insurance Agency therefore has information about SA spells of unemployed from day 2 and of employed from day 15. In order not to introduce selection bias for unemployment, we only included SA spells > 14 days. During the study period, there was an upper limit of 914 days for SA spells. In the Swedish diagnosis-specific guidelines for SA duration regarding knee osteoarthritis, the recommendations vary depending on pain severity, physical work demands, and type of treatment, e.g., surgery or not, as well as comorbidity. In most cases, partial SA is recommended for a short time, however in some severe situations for up to six months [18–20]. Additionally, all Swedish residents aged 19–64 can be granted disability pension if they have long-term or permanently reduced work incapacity due to disease or injury. Both SA and DP benefits can be granted for full-time (100 %) or part-time (75 %, 50 %, or 25 %) of ordinary work hours. SA benefits cover 80 % and DP covers 64 % of lost income, both up to a defined ceiling level of income.

### Potential predictors

Information on potential predictors was obtained from MiDAS and by linkage to four other nationwide registers at the individual level, using the unique personal identification number. From MiDAS: prior SA, extent of SA (100 %, 75 %, 50 %, or 25 %) and employment status at the start of the spell. From Statistics Sweden: socio-demographic variables age, sex, country of birth, educational level, occupational sector, marital status, and geographical area was obtained from the Longitudinal integration database for health insurance and labor market studies (Swedish abbreviation: LISA) [21]. From the National Board of Health and Welfare, we obtained information on specialized (inpatient and specialized outpatient) healthcare from the inpatient and outpatient registers [22] and information on prescription drug dispenses (ATC codes and date of dispenses) from the prescribed drug register [23] in the 365 days preceding start date of the SA spell as well as death dates from the cause of death register [24].

At first, 130 predictors were chosen for manual curation from the registers, based on their feasibility for clinical implementation and scope for association to the outcome. This curation was done by the professors and senior researchers through consensus-seeking discussion and though analyses of their predicitve value. Most variables were excluded due to covering the same aspects, such as previous number of SA net days or SA gross days. The majority of predictors were redundant and/or expected to be highly collinear with at least one other predictor, e.g., net and gross days of SA. For predictors that were hierarchically related, e.g., multi-morbidity and cause-specific morbidity, general and cause-specific hospitalization, etc., we consistently chose the general/coarse variables over the specific ones as it would improve both parsimony and the scope for implementation of the model in clinical practice. From the initial set, 14 predictors remained after curation, all categorical: Age (categorized into 18–34, 35–40, 41–50, 51–57, 58–64), Sex (woman/man), Geographical region (categorized into five groups: North, Mid, West, South Sweden, and Stockholm/Gotland), Educational level (elementary (≤ 9 years including missing), high school (10–12 years), university/college (> 12 years)), Family situation (composite four-level variable of ‘Married/living with partner’ yes/no, and ‘living with children < 18 years old’ yes/no), Country of birth (“Sweden”, “Non-Swedish Nordic country”, “Non-Nordic EU25 country”, “Non-EU country including missing”), Number of SA days in the 12 months preceding baseline (None, < 3 months, 3–6 months, or > 6 months), Number of specialized outpatient healthcare visits 12 months before baseline (0, 1–2, > 2), Number of inpatient days 12 months preceding baseline (0, 1–2, > 2), Extent of SA at start of the SA spell (25 %, 50 %, 75 %, or 100 %), Partial disability pension at baseline (yes/no), Employment status at baseline (“Employed/Student”, “Parental leave”, “Unemployed”), Multi-morbidity (defined as > 1 drug dispenses of prescribed medication of at least 3 different ATC codes (at 1-digit level) in the 12 months preceding baseline) (yes/no), and Specialized healthcare at start of SA (yes/no). Baseline here refers to the start date of the SA spell. When calculating in- and outpatient visits, we excluded any visits due to full-term uncomplicated deliveries (ICD-10 O80), counseling, general medical advice and screening (ICD-10 codes Z00-Z99, with the exception of Z73.0 coding for ‘burnout syndrome’ which was included).

### Statistical analysis

Piecewise constant hazards regression models were fitted to predict the duration of the SA spell, using the ‘pch’ package (version 1.3) in R [25]. The model was specified to use 20 time intervals. The main outcome variable was SA spell duration in number of days, defined as the total number of gross days from the start date of the spell to the last day with SA benefit, excluding days for which no SA benefit was paid from the Social Insurance Agency, which is sometimes the case during already planned vacations. Whenever the duration exceeded a total of 1000 SA days it was cut at day 1000 (n = 197 SA spells) to avoid modeling extreme outliers. We also registered if the SA spell ended due to being granted disability pension, emigration, or death. If it ended in disability pension, the SA spell was coded as exceeding 1000 days.

### Development of the model

The data was split into development (70 %) and validation (30 %) data, using random sampling without replacement. Variable selection was performed in the development dataset, and the performance of the models was evaluated by predicting the duration of the SA spell on observations in the validation data and comparing it to the observed durations. It was supposed that it would not be possible for the general practitioner to have time to ask about and register more than six factors during the consultation. Thus, to facilitate clinical use of the model, it was a pre-specified aim to construct a parsimonious model with no more than 9 predictors. Three of these (age, gender, and geographical region), were a priori decided to surpass variable selection and be forced into the model – as they could be included automatically in the model at the primary healthcare centers. The other six variables were chosen based on their ranking of log-likelihood loss when excluding them one at a time from the full model including the 14 predictors obtained after curation, as described above.

### Evaluation of the model

Model comparisons of goodness-of-fit were done using the Akaike and Bayesian information criteria (AIC and BIC). Calibration-in-the-large was assessed visually by a quantile-quantile plot of the uniformity of the cumulative distribution function. The overall discriminatory capacity was assessed with the *c*-statistic [26, 27]. Corresponding 95 % confidence intervals (CI) for *c* were obtained using bootstrap resampling (x1000).

The ability of the model to predict SA spells lasting longer than 90, 180, or 365 days, respectively, was assessed by dichotomizing the observed SA durations at 90, 180, or 365 days respectively, and calculating the area under the receiver operating characteristics (ROC) curve (AUC) across predicted probability thresholds, using DeLong’s method for computing 95 % CIs [28]. Model calibration for each of the three binary outcomes was assessed visually by plotting the smoothed calibration curve using natural splines with three degrees of freedom.

**Fig. 1:**
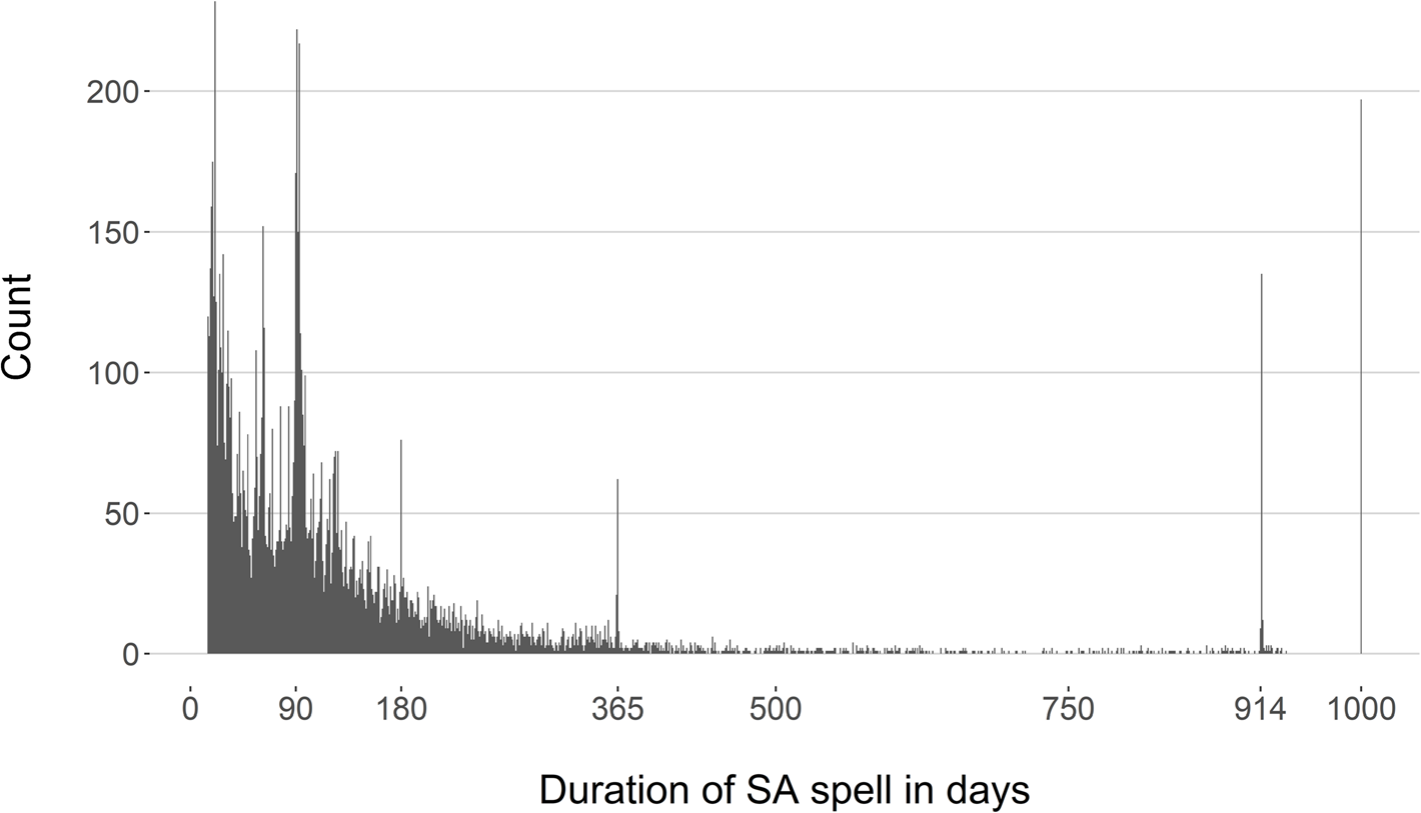
Histogram over SA spells of different durations, in days

All statistical analyses were done in R version 3.4.3 [29] with packages ‘pch’, ‘pROC’, ‘e1071’, and ´Hmisc’. Figure 1 was produced using the ggplot2 and ggthemes packages [30].

## Results

A total of 12,098 new SA spells > 14 days due to M17 from 10,656 individuals were identified for the 2.5-year period and included in the analyses. Their characteristics are tabulated in Table 1.

**Table 1:** Descriptive statistics of all the included sickness absence (SA) spells due to M17

Characteristics	Number (%)
Total number of SA spells	12,098 (100)
Total number of unique individuals	10,656 (100)
Individuals with more than one such SA spell	1280 (12)
Duration of the SA spell	
>14 and ≤ 90 days	5707 (47)
>90 days	6391 (53)
>180 days	2578 (21)
>365 days	922 (8)
>999 days	197 (2)
Situation at end of SA spell	
Death	18 (0)
Disability pension	190 (2)
The individual was > 65 years old	286 (2)
Emigrated from Sweden	< 8 (0)
Return to work or other reason	11,601 (96)

The distribution of the SA duration was positively skewed, with a group of outlier observations with very long SA (Fig. 1). The mode of SA duration was 21 days (for *n* = 232 spells), with a median of 92 days and interquartile range of 45–161 days (Table 1; Fig. 1).

Predictors tabulated in the development and validation datasets are shown in Table 2 (the corresponding table for the 14 predictors in the full model can be found in Supplementary Table 1). The final model contained nine predictors, as pre-specified. Apart from age, sex, and geographical region, the predictors showing greatest log-likelihood loss were occupational status, educational level, specialized healthcare at start of SA, number of visits to specialized outpatient clinics the previous 12 months, number of days in inpatient healthcare the previous 12 months, and number of days with SA benefits during the previous 12 months before start date.

**Table 2:** Tabulation of the final included baseline predictors in the development and validation data sets

Predictor	Development data n SA spells (%)		Validation data n SA spells (%)			
Sex						
Women	4654 (55.0)		1970 (54.5)			
Men	3814 (45.0)		1652 (45.5)			
Age groups						
18–30 years	62 ( 0.7)		15 ( 0.4)			
31–40 years	241 ( 2.8)		100 ( 2.8)			
41–50 years	1350 (15.9)		520 (14.3)			
51–57 years	2580 (30.5)		1171 (32.3)			
58–64 years	4235 (50.0)		1824 (50.2)			
Geographical region						
North	1228 (14.5)		522 (14.4)			
Middle	1222 (14.4)		527 (14.5)			
Stockholm/Gotland	1483 (17.5)		614 (16.9)			
West	2628 (31.0)		1105 (30.4)			
South	1907 (22.5)		862 (23.7)			
Educational level (years)						
Elementary school (≤ 9 years)	1839 (21.7)		814 (22.4)			
High School (10–12 years)	4664 (55.1)		2042 (56.3)			
College/university (> 12 years)	1965 (23.2)		774 (21.3)			
Number of inpatient healthcare days in the 12 months before start date of the SA spell^1^						
0	6507 (76.8)		2804 (77.2)			
1–2	872 (10.3)		393 (10.8)			
>2	1089 (12.9)		433 (11.9)			
Number of SA days in the 12 months before start date of the SA spell						
0	5532 (65.3)		2336 (64.4)			
(0–90]	2070 (24.4)		901 (24.8)			
(90–180]	356 ( 4.2)		144 ( 4.0)			
(180–366]	510 ( 6.0)		249 ( 6.9)			
Specialized outpatient healthcare in the 12 months before start date of the SA spell^1^						
0	1569 (18.5)		691 (19.0)			
1–2	3549 (41.9)		1531 (42.2)			
>2	3350 (39.6)		1408 (38.8)			
Employment status at baseline (SA start date)						
Employed/student	7905 (93.4)		3393 (93.5)			
Parental leave	13 ( 0.2)		< 8 ( 0.1)			
Unemployed	550 ( 6.5)		235 ( 6.5)			
Specialized healthcare at start of the SA spell						
No	3265 (38.6)		1439 (39.6)			
Yes	5203 (61.4)		2191 (60.4)			

1 = Excluding healthcare with O80 and Z-codes Z00-Z99 except Z73.0

SA = sickness absence

### Predictive performance of the model

**Fig. 2:**
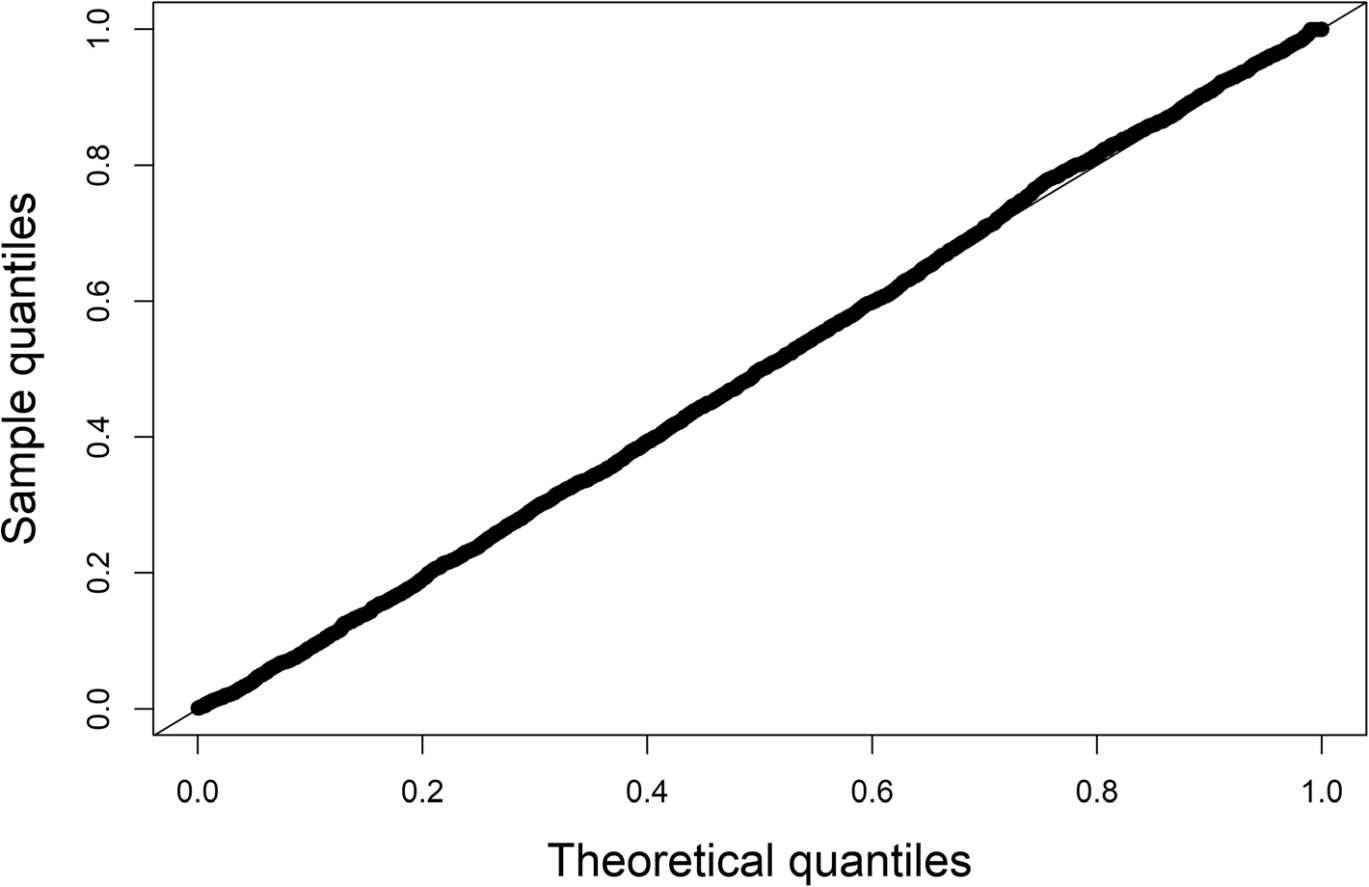
Q-Q plot of model calibration for predicted SA spell duration

**Fig. 3:**
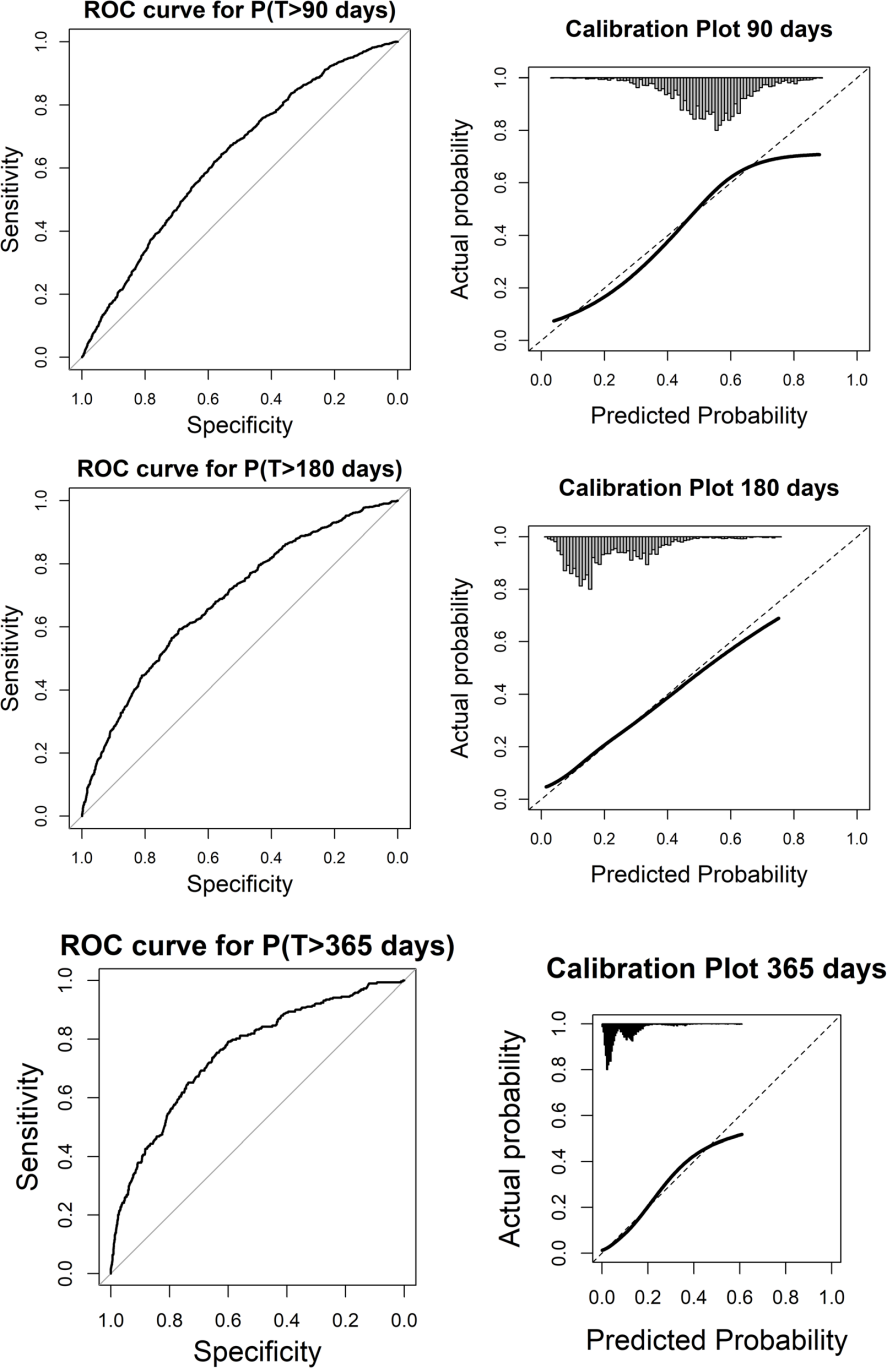
ROC curves and Calibration Plots for the predictive models of SA spells of >90 days, >180 days, and >365 days, respectively

Both the AIC and BIC were slightly lower in the final rather than in the full model (Table 3), indicating a better fit of the more parsimonious model. Calibration-in-the-large was good, as shown in Fig. 2, indicating that the model was correctly specified. The overall discriminatory capacity on individual level was low, with *c*-statistic = 0.53 (95 % CI 0.52–0.54) (Table 4). Binary predictions of risk of long-term SA showed poor performance for relatively short-term outcomes (> 90 days, > 180 days) but good discriminatory ability for predicting long SA durations (> 365 days), with *c*-statistic = 0.63 (95 % CI 0.61–0.65) for > 90 days, 0.69 for > 180 days, and 0.75 (0.72–0.78) for > 365 days (Table 4; Fig. 3). Calibration for all binary outcomes, as shown in Fig. 3, was satisfactory. Beta estimates for each predictor at each of the 20 time points can be found in Supplementary Tables 2 and 3.

**Table 3:** Goodness of fit of the full vs. the final models

	Full model (14 predictors)	Final model (9 predictors)
N observations	8468	8468
N free parameters	620	440
Log-likelihood	-47,252	-47,413
AIC	95,740	95,702
BIC	100,093	98,787

**Table 4:** Predictive performance of the model. *C*-statistic for continuous outcome in survival analysis, and corresponding metric AUC for dichotomized outcomes at predefined cutoffs of SA duration

	*C-*statistic (95 % CI)
Continuous outcome	
Overall	0.53 (0.52–0.54)
Dichotomous outcomes	
SA > 90 days	0.63 (0.61–0.65)
SA > 180 days	0.69 (0.66–0.71)
SA > 365 days	0.75 (0.72–0.78)

## Discussion

This is, as far as we know, the first prediction model of SA duration for knee osteoarthritis, and further improvements to the model may well be possible. However, we were able to achieve satisfactory discrimination between long-lasting (> 180 and > 365 days) and expected length-SA spells using the model [18]. The model was built on readily available real-world data regarding socio-demographic and patient history information, some of which have been previously found to be predictive of duration of SA from any cause. It would be of future interest to additionally assess general risk factors for osteoarthritis such as obesity, heredity/genes, and occupational exposure to kneeling, as well as prognostic factors for clinical progression such as baseline osteoarthritis severity and other knee lesions [31], as potentially stronger predictors of SA duration in knee osteoarthritis. Previous research on future trajectories of SA/DP among those with an initial SA-spell due to osteoarthritis (all types) shows that trajectories of future SA/DP are quite heterogeneous [32]. However, those individuals who belonged to trajectories that had not reached zero SA or DP days after a 13-month follow-up were distinct on a number of characteristics: they were older, more likely to be born outside EU25, and more likely to have had SA due to a mental diagnosis prior to the SA due to osteoarthritis [32]. That the individuals belonging to trajectories that did not reduce their SA/DP to 0 days per month after 13 months were distinct from those belonging to trajectories that did, supports our finding that we had greater success at predicting the long SA spells (> 180 and > 365 days, respectively).

There were certain predictors that were in the larger model, based on hypothesized or previously demonstrated association with duration of SA, but which were not included in the final model due to lower log-likelihood loss when excluding them, such as occupational sector or birth country. Comparing our final model to the first larger model with 14 predictors, the final model showed very similar discriminatory ability, and only a slightly lowered AIC and BIC. The final model is thus comparable in performance to the larger model, but with the advantage of being more parsimonious and easier to implement in clinical practice.

We found that of all the new SA spells due to knee osteoarthritis > 14 days in Sweden, 53 % became > 90 days. This is in line with the previously reported proportion of 53 % for physician-certified SA spells > 8 days for knee osteoarthritis issued during the years 2009–2010 in Southern Sweden [33]. These numbers thus appear to be stable geographically and temporally over a four-year span. If social insurance policies and sickness certification practices remain constant, our model would be expected to perform as reported for our internal validation in the Swedish setting. For clinical use of our model in non-Swedish settings, external validation is strongly recommended prior to use.

The prediction model can be used by physicians in consultations with patients who recently were or are about to be SA certified – so far it is used in more than 20 healthcare units. The physician inserts information about the patient in the computerised prediction model, and optionally specifies the duration of sickness absence (> 90 days, > 180 days or > 365 days) for which to obtain a probability. The model outputs a probability score that can be used by the practitioner for early identification of patients with high risk for long-term SA. The model’s output can also be used as a basis for discussion with the patient regarding their sickness absence, and how to handle possible high risk of long-term SA in order to promote his/her return to work.

Early identification of SA spells that are at high risk of becoming long-term means a possibility that resources and support, such as rehabilitation coordinators, supported return to work, etc. can more accurately be targeted to those with greater need of them. The model does not identify which particular measures will be more or less useful in preventing the SA spell becoming long. For that other types of studies are warranted. The type of treatment given can influence levels of symptoms patient experiences and functional limitations due to osteoarthritis [34, 35], which may of course matter for SA duration – however, this is beyond the scope of this study.

Strengths of our work include the population-based design with full coverage from the used nationwide registers, that is, all new SA spells were included, not a sample, and data linkage could be performed for all, data were of high quality, not affected by self-reports or drop-outs. Additionally, in the population of Sweden, there is high employment frequency among both women and men, as well as in higher ages where osteoarthritis is prevalent [4]. The use of nationwide high-quality registers also ensures near full completeness of follow-up [24, 36]. Unlike our study, most studies on SA duration have been performed in selective populations of certain occupations, and/or were based on voluntary study-participation with drop-outs, which hampers external validity.

The register-based approach also limits the resulting model in some regards. We had no information on smoking or self-rated health, which have previously been found to be predictive of SA spells lasting > 90 days [37]. Nor could we assess the predictive value of variables related to osteoarthritis severity, as such information was not available in the used registers. Our model is internally validated under the conditions applying to the Swedish healthcare and social insurance systems. Currently, temporal validation is ongoing in several separate healthcare centers in geographically separated counties in Sweden.

## Conclusions

Using the model of sickness absence (SA) duration for people on SA due to knee osteoarthritis, it was possible to discriminate long-term SA spells with moderate precision, although shorter-term individual-level discrimination was low. As there are currently no other prediction models for this purpose, our model is a first step towards improving handling of SA spells and redirecting rehabilitation efforts for patients on SA with knee osteoarthritis. External validation of the model in other countries is encouraged to assess the scope for generalization.

## Supplementary Information

**Additional file 1:**
