# Supplementary material for: Predicting the duration of sickness absence due to knee osteoarthritis: a prognostic model developed in a population-based cohort in Sweden
Source: BMC Musculoskelet Disord. 2021 Jul 2;22:603. doi: 10.1186/s12891-021-04400-8 (PMC8254363; doi:10.1186/s12891-021-04400-8)
Supplement: Supplementary file 1 — Additional file 1: [file 12891_2021_4400_MOESM1_ESM.pdf]

Predicting the duration of sickness absence due to knee osteoarthritis: A prognostic model developed in a population-based cohort in Sweden

Dr. Johanna Holm, Ph.D., Dr. Paolo Frumento, Ph.D., Gino Almondo, MSc., Dr. Katalin Gémes, Ph.D., Prof. Matteo Bottai, Sc.D., Prof. Kristina Alexanderson, Ph.D., Dr. Emilie Friberg, Ph.D., Dr. Kristin Farrants, Ph.D.

Supplementary table 1. Distribution of the full fourteen predictors, sector and occupation, among unique individuals and number of sickness absence (SA) spells

Demographics	N Of Individuals (%)	N Of Sickness Absence Spells (%)
Sex		
Women	5795 (54.4)	6632 (54.8)
Men	4864 (45.6)	5466 (45.2)
Age group		
18-30 years	75 (0.7)	77 (0.6)
31-40 years	322 (3.0)	341 (2.8)
41-50 years	1675 (15.7)	1870 (15.5)
51-57 years	3309 (31.0)	3751 (31.0)
58-64 years	5278 (49.5)	6059 (50.1)
Geographical region of Sweden		
North	1512 (14.2)	1750 (14.5)
Middle	1541 (14.5)	1749 (14.5)
Stockholm/Gotland	1851 (17.4)	2097 (17.3)
West	3306 (31.0)	3733 (30.9)
South	2449 (23.0)	2769 (22.9)
Educational level (years)		
Elementary school (≤9 Years)	2293 (21.5)	2653 (21.9)

High school (10-12 Years)	5935 (55.7)	6706 (55.4)
College/university>12 Years	2431 (22.8)	2739 (22.6)
Family situation		
Married/cohabitant		
Living with children <18 years old	3075 (28.8)	3437 (28.4)
Not living with children <18 years	3851 (36.1)	4404 (36.4)
Single/divorced/separated/widowed		
Living with children <18 years old	746 (7.0)	855 (7.1)
Not living with children <18 years	2987 (28.0)	3402 (28.1)
Country of birth		
Sweden	9366 (87.9)	10633 (87.9)
Non-Swedish Nordic country	517 (4.9)	580 (4.8)
Non-Nordic EU25 country	176 (1.7)	199 (1.6)
Non-EU25 country	600 (5.6)	686 (5.7)
Number of SA days in the 12 months preceding the start date of SA spell		
0	7760 (72.8)	7868 (65.0)
(0-90]	2041 (19.1)	2971 (24.6)
(90-180]	474 (4.4)	759 (6.3)
(180-366]	384 (3.6)	500 (4.1)
Outpatient healthcare visits[1] in 12 months preceding the start date of SA spell		

0	2072 (19.4)	2260 (18.7)
1- 2	4571 (42.9)	5080 (42.0)
>2	4016 (37.7)	4758 (39.3)
Inpatient healthcare days[1], in the 12 months preceding the start date of SA spell		
0	8454 (79.3)	9311 (77.0)
1-2	1171 (11.0)	1265 (10.5)
>2	1034 (9.7)	1522 (12.6)
Extent of SA at initiation of the SA spell		
25%	292 (2.7)	364 (3.0)
50%	1398 (13.1)	1677 (13.9)
75%	370 (3.5)	417 (3.4)
100%	8599 (80.7)	9640 (79.7)
Partial disability pension at start of the SA spell		
No	9633 (90.4)	10962 (90.6)
Yes	1026 (9.6)	1163 (9.6)
Employment status at start of the SA spell		
Employed/student	9936 (93.2)	11298 (93.4)
Parental leave	15 (0.1)	15 (0.1)
Unemployed	708 (6.6)	785 (6.5)
Sector		

None	655 (6.1)	715 (5.9)
Public	4041 (37.9)	4653 (38.5)
Private	5963 (55.9)	6730 (55.6)
Occupation		
No information	386 (3.6)	430 (3.6)
Blue collar	6808 (63.9)	7782 (64.3)
White collar	3465 (32.5)	3886 (32.1)
Multimorbidity[2]		
No	6429 (60.3)	7182 (59.4)
Yes	4230 (39.7)	4916 (40.6)
Specialist healthcare at start of the SA spell		
No	4075 (38.2)	4704 (38.9)
Yes	6584 (61.7)	7394 (61.1)

1 = Excluding healthcare with Z-codes and O80

2 = More than one expenditure of prescribed drugs from at least three different ATC codes, during the 12 months preceding the start date of the SA spell

Supplementary Table 2. Beta estimates (95% CI) from the final model fit, for the intercept and each parameter, fitted at each of the 20 intervals, presented for the first 10 intervals regarding sickness absence (SA) spells due to knee osteoarthritis

Predictors	Betas by piecewise intervals 1-10									
	1	2	3	4	5	6	7	8	9	10
**Intercept**	-2.89 (0.82)	-3.98 (1.01)	-3.01 (0.67)	-3.13 (0.86)	-4.03 (0.88)	-4.03 (1.04)	-3.81 (1.21)	-3.88 (1.52)	-4.4 (1.49)	-15.56 (114.64)
**Geographical region**										
Middle	Reference									
North	0.03 (0.38)	0.21 (0.39)	0.22 (0.35)	0 (0.42)	0.04 (0.37)	0.15 (0.38)	0.13 (0.34)	-0.16 (0.34)	-0.19 (0.39)	-0.17 (0.38)
Stockholm/Gotland	-0.01 (0.38)	0.18 (0.37)	0.21 (0.34)	0.52 (0.37)	0.11 (0.35)	0.17 (0.36)	0.21 (0.31)	0.09 (0.31)	0.23 (0.33)	-0.04 (0.35)
South	0.25 (0.34)	0.56 (0.34)	0.07 (0.33)	0.17 (0.38)	0.25 (0.33)	0.2 (0.35)	-0.31 (0.33)	-0.35 (0.31)	0.11 (0.32)	-0.03 (0.32)
West	-0.06 (0.34)	0.29 (0.33)	0.22 (0.31)	0.39 (0.35)	0.25 (0.31)	0.08 (0.33)	-0.09 (0.3)	-0.13 (0.28)	-0.04 (0.31)	-0.08 (0.31)
**Sex**										
Female	Reference									
Male	-0.16 (0.21)	0.02 (0.2)	-0.03 (0.19)	0.05 (0.21)	0 (0.2)	0.32 (0.21)	0.17 (0.2)	0.25 (0.19)	0.1 (0.2)	0.16 (0.21)
**Age groups**										
17-29 years	Reference									
30-39 years	-0.62 (0.83)	0.16 (1)	-0.64 (0.68)	-0.72 (0.85)	-0.16 (0.9)	-0.42 (1.02)	-0.2 (1.25)	0.09 (1.58)	-0.59 (1.62)	11.84 (114.65)
40-49 years	-0.92 (0.73)	-0.14 (0.95)	-1.02 (0.59)	-1.28 (0.77)	-0.73 (0.83)	-0.95 (0.94)	-1.01 (1.17)	-0.64 (1.5)	-0.48 (1.43)	11.85 (114.64)
50-56 years	-0.99 (0.72)	-0.58 (0.95)	-1.45 (0.58)	-1.62 (0.76)	-0.99 (0.82)	-1.36 (0.93)	-0.93 (1.15)	-0.68 (1.49)	-0.54 (1.42)	11.71 (114.64)
57-63 years	-1.54 (0.72)	-0.79 (0.94)	-1.8 (0.58)	-1.87 (0.75)	-1.42 (0.82)	-1.53 (0.92)	-0.85 (1.14)	-0.65 (1.48)	-0.62 (1.42)	12.08 (114.64)
**Employment status**										
Employed/student	Reference									
Unemployed	-0.35 (0.54)	-1.13 (0.67)	-0.31 (0.46)	-0.86 (0.61)	-0.49 (0.47)	-0.27 (0.43)	0.87 (0.29)	-0.95 (0.57)	-0.03 (0.4)	-0.13 (0.44)
Parental leave	1.21 (1.36)	0.77 (1.38)	0.72 (1.14)	-11.28 (37.69)	0.6 (1.91)	-11.44 (45.23)	-11.11 (38.6)	-11.18 (36.34)	-10.91 (39.18)	-11.86 (118.42)
**Educational level (years)**										
Elementary school (≤9 years)	-0.29 (0.28)	-0.01 (0.25)	0.05 (0.24)	-0.09 (0.26)	-0.06 (0.25)	-0.25 (0.28)	-0.15 (0.27)	-0.22 (0.26)	0.08 (0.26)	-0.13 (0.26)
High School (10-12 years)	Reference									
College/university (>12 years)	-0.12 (0.26)	0.08 (0.24)	0.16 (0.23)	-0.13 (0.27)	0.06 (0.24)	0.23 (0.24)	0.53 (0.22)	0.41 (0.22)	0.59 (0.22)	0.21 (0.24)
**Gross days of SA during the year before**										
>180 and ≤366	-2.53 (1.99)	-0.43 (0.6)	-0.91 (0.77)	-0.86 (0.76)	-0.72 (0.63)	-1.11 (0.72)	-0.98 (0.65)	-0.79 (0.55)	-0.89 (0.61)	-2.44 (1.41)
>90 and ≤180	-0.14 (0.51)	-0.04 (0.44)	-0.63 (0.57)	-0.13 (0.48)	-0.4 (0.48)	-0.21 (0.45)	-0.41 (0.45)	-1.13 (0.56)	-1.05 (0.56)	-0.55 (0.45)

>0 and ≤90	0.2 (0.24)	0.1 (0.23)	0.07 (0.22)	0.28 (0.23)	0.1 (0.23)	-0.1 (0.26)	-0.12 (0.25)	-0.24 (0.23)	-0.24 (0.24)	-0.17 (0.25)
0	Reference									
**The SA spell was initiated in the primary healthcare**										
Yes	Reference									
No	0.36 (0.24)	0.6 (0.22)	0.41 (0.22)	0.53 (0.25)	0.7 (0.22)	0.35 (0.24)	-0.36 (0.24)	-0.1 (0.22)	-0.49 (0.25)	-1.13 (0.3)
**Number of outpatient care visits during the 365 days before the SA spells**										
0	Reference									
1-2	-0.28 (0.25)	-0.09 (0.24)	-0.27 (0.25)	-0.42 (0.27)	-0.17 (0.25)	-0.26 (0.31)	0.08 (0.34)	-0.6 (0.28)	-0.07 (0.35)	0.16 (0.38)
>2	-0.79 (0.32)	-0.45 (0.29)	-0.57 (0.27)	-0.61 (0.3)	-0.55 (0.29)	-0.18 (0.31)	0.05 (0.35)	-0.39 (0.28)	0.07 (0.35)	0.05 (0.39)
**Number of inpatient healthcare days in the 365 days before the SA spell**										
0	Reference									
1-2	-1.08 (0.62)	-0.74 (0.48)	-0.85 (0.47)	-0.65 (0.49)	-0.46 (0.41)	-0.25 (0.38)	-0.06 (0.29)	-0.04 (0.3)	0.14 (0.27)	0.07 (0.28)
>2	-0.43 (0.44)	-0.32 (0.36)	-0.53 (0.4)	0.06 (0.34)	0.05 (0.34)	0.42 (0.31)	0.19 (0.29)	0.59 (0.27)	0.27 (0.3)	0.33 (0.29)

Supplementary table 3. Beta estimates (95% CI) from the final model fit, for the intercept and each parameter, fitted at each of the 20 intervals, presented for the last 10 intervals regarding sickness absence (SA) spells due to knee osteoarthritis.

**Predictors**	**Betas by piecewise intervals 11-20**									
	**11**	**12**	**13**	**14**	**15**	**16**	**17**	**18**	**19**	**20**
**Intercept**	-3.09 (1.62)	-4.73 (1.68)	-16.84 (178.91)	-4.74 (2.18)	-4.65 (2.18)	-3.51 (1.56)	-4.8 (1.57)	-3.5 (1.42)	-5.34 (0.46)	-5.66 (0.2)
**Geographical regions**										
Middle	Reference									
North	-0.11 (0.32)	0.17 (0.34)	0.04 (0.37)	-0.03 (0.36)	-0.2 (0.36)	-0.33 (0.39)	-0.06 (0.35)	0.08 (0.37)	0.31 (0.38)	-0.09 (0.17)
Stockholm/Gotland	-0.19 (0.31)	-0.04 (0.34)	0.04 (0.35)	-0.27 (0.36)	-0.15 (0.34)	0.07 (0.34)	-0.15 (0.36)	0.29 (0.36)	0.37 (0.38)	0 (0.17)
South	-0.41 (0.29)	-0.27 (0.32)	-0.06 (0.33)	-0.21 (0.32)	-0.1 (0.3)	0.05 (0.32)	0 (0.32)	0.24 (0.34)	0.2 (0.38)	-0.15 (0.15)
West	-0.53 (0.28)	-0.25 (0.3)	0.05 (0.3)	-0.05 (0.3)	-0.34 (0.3)	0 (0.31)	-0.15 (0.31)	0.09 (0.33)	0.43 (0.34)	-0.01 (0.16)
**Sex**										
Female	Reference									
Male	0.13 (0.19)	0.12 (0.2)	-0.02 (0.2)	0.04 (0.2)	-0.07 (0.2)	0.24 (0.19)	0.14 (0.2)	0.14 (0.2)	0.23 (0.22)	0.11 (0.1)
**Age groups**										
17-29 years	Reference									
30-39 years	-0.34 (1.75)	0.28 (1.8)	11.66 (178.92)	0.49 (2.25)	-0.11 (2.31)	-0.4 (1.6)	0.2 (1.67)	-1.8 (1.57)	-0.69 (1.04)	0.09 (0.25)
40-49 years	-0.7 (1.57)	0.37 (1.63)	11.87 (178.91)	0.05 (2.17)	0.06 (2.16)	-0.83 (1.52)	-0.11 (1.53)	-2.03 (1.4)	-0.41 (0.32)	-0.03 (0.12)

50-56 years	-0.21 (1.54)	0.31 (1.62)	12.05 (178.91)	0.26 (2.16)	0.29 (2.14)	-0.76 (1.5)	0.1 (1.52)	-2.05 (1.39)	-0.03 (0.23)	0.03 (0.11)
57-63 years	-0.08 (1.54)	0.44 (1.62)	12.06 (178.91)	0.44 (2.15)	0.3 (2.14)	-0.72 (1.5)	0.21 (1.51)	-1.87 (1.39)	0 (0)	0 (0)
**Employment status**										
Employed/student					Reference					
Unemployed	0.35 (0.35)	-0.1 (0.42)	-0.17 (0.44)	-0.26 (0.43)	-0.29 (0.42)	-0.87 (0.5)	-0.76 (0.43)	-1.32 (0.5)	-1.16 (0.37)	-0.14 (0.1)
Parental leave	-10.6 (34.57)	-10.99 (40.19)	0.51 (2.23)	0.31 (2.25)	0.81 (2.35)	0.1 (1.33)	-9.99 (76.14)	-11.18 (201.95)	-11.69 (203.46)	-0.42 (0.19)
**Educational level (years)**										
Elementary school (≤9 years)	-0.01 (0.24)	-0.24 (0.27)	-0.24 (0.26)	-0.18 (0.25)	0.05 (0.23)	-0.27 (0.24)	0.22 (0.22)	-0.22 (0.24)	-0.32 (0.26)	-0.1 (0.11)
High School (10-12 years)					Reference					
College/university (>12 years)	0.19 (0.23)	0.33 (0.24)	0.26 (0.23)	0.17 (0.24)	0.03 (0.27)	0.19 (0.25)	-0.06 (0.29)	0.11 (0.27)	-0.11 (0.32)	0 (0.15)
**Gross days of SA during the 365 days before start of the SA spell**										
>180 and ≤366	-1.26 (0.66)	-0.97 (0.59)	-1.28 (0.64)	-0.84 (0.53)	-1.1 (0.59)	-0.41 (0.42)	-0.16 (0.37)	-0.44 (0.41)	-0.45 (0.37)	-0.03 (0.14)
>90 and ≤180	-0.77 (0.45)	-0.73 (0.46)	-0.44 (0.42)	-0.17 (0.35)	0 (0.36)	0.17 (0.33)	0.01 (0.38)	0.22 (0.36)	0.06 (0.42)	-0.17 (0.13)
>0 and ≤90	-0.2 (0.23)	-0.19 (0.24)	-0.18 (0.24)	-0.13 (0.24)	-0.06 (0.24)	-0.27 (0.25)	-0.07 (0.24)	-0.03 (0.23)	-0.23 (0.26)	0.18 (0.14)
0					Reference					
**The SA spell was initiated in the primary healthcare**										
Yes					Reference					
No	-1.81 (0.32)	-0.87 (0.26)	-0.99 (0.26)	-1.14 (0.26)	-0.89 (0.22)	-0.87 (0.21)	-0.66 (0.21)	-0.13 (0.22)	-0.3 (0.25)	-0.04 (0.11)
**Number of outpatient health care visits during the 365 days before the SA spells**										
0					Reference					
1-2	0.19 (0.38)	0.08 (0.36)	0.53 (0.41)	0.51 (0.38)	0.26 (0.31)	-0.05 (0.28)	0.07 (0.29)	0.2 (0.26)	0.01 (0.28)	0.04 (0.13)
>2	0.14 (0.39)	0.22 (0.37)	0.56 (0.42)	0.41 (0.4)	0.24 (0.33)	-0.1 (0.29)	0.2 (0.3)	0.16 (0.3)	-0.05 (0.32)	-0.03 (0.14)
**Number of inpatient healthcare days in the 365 days before the SA spell**										
0					Reference					
1-2	0.15 (0.26)	-0.04 (0.3)	0.25 (0.26)	0.19 (0.28)	0.26 (0.29)	0.29 (0.3)	0.16 (0.34)	0.24 (0.38)	0.09 (0.45)	-0.06 (0.2)
>2	0.49 (0.26)	0.36 (0.3)	0.41 (0.31)	0.28 (0.3)	0.07 (0.31)	-0.07 (0.34)	-0.26 (0.34)	-0.16 (0.34)	-0.29 (0.35)	-0.07 (0.12)
